# The Mediterranean Diet and Cerebrovascular Risk Factors: A Lifeline for Vascular Health—Narrative Review

**DOI:** 10.3390/nu18081273

**Published:** 2026-04-17

**Authors:** Gaetano Pacinella, Andrea Salvo, Carlo Domenico Maida, Mario Daidone, Stefania Scaglione, Anna Maria Ciaccio, Antonino Tuttolomondo

**Affiliations:** 1 Department of Promoting Health, Maternal-Infant, Excellence and Internal and Specialised Medicine (PROMISE) G. D’Alessandro, University of Palermo, 90127 Palermo, Italybruno.tuttolomondo@unipa.it (A.T.); 2 Internal Medicine and Stroke Care Ward, Policlinico “P. Giaccone”, 90127 Palermo, Italy; 3 Department of Internal Medicine, S. Elia Hospital, 93100 Caltanissetta, Italy

**Keywords:** Mediterranean diet, MedDiet, ischemic stroke, acute cerebrovascular syndrome, cerebrovascular risk

## Abstract

Ischemic stroke and its chronic cerebrovascular complications represent significant public health challenges with considerable societal impact. Consequently, healthcare initiatives worldwide are placing greater emphasis on preventing and lowering cerebrovascular risk. Alongside medical therapies, it is now widely recognized that modifying risk factors—many of which are controllable—can substantially reduce the probability of acute cerebrovascular events, up to 33% according to data from trials such as PREDIMED. This modification is often achievable through dietary interventions such as the Mediterranean diet (MedDiet), which positively influences vascular health. The MedDiet, long established as the traditional eating pattern in Mediterranean regions, is an effective means of counteracting factors that jeopardize vascular health and elevate the risk of acute events. To date, there are no narrative reviews that have addressed the impact of the Mediterranean diet on cerebrovascular risk and the consequences of acute cerebrovascular events in terms of disability and neurological functional recovery, focusing on how individual components of the MedDiet, at the molecular level, contribute to the prevention of acute vascular episodes, paving the way for new approaches in the treatment of cerebrovascular patients.

## 1. Introduction

Cerebrovascular disorders, particularly those with ischemic pathogenesis such as ischemic stroke due to vascular occlusion or small vessel disease, continue to be a major contributor to death and lasting disability across the globe, largely fueled by modifiable factors like high blood pressure, dyslipidemia, excess weight, and chronic inflammation. More recently, the focus has shifted to how eating habits can influence and reduce vascular risk. In this context, the Mediterranean diet (MedDiet) has gained recognition as a nutritional approach strongly supported by scientific data for its heart- and brain-protective effects. This diet emphasizes abundant consumption of fruits, vegetables, whole grains, legumes, nuts, extra-virgin olive oil, along with moderate amounts of fish and red wine, all of which supply numerous antioxidants and anti-inflammatory agents [1].

This manuscript underscores the increasing acknowledgment of the Mediterranean diet as an effective non-drug approach to maintaining cerebrovascular health throughout life. The purpose of this article is to compile and present recent findings on how the MedDiet can lower cerebrovascular risk, while highlighting the pressing need to incorporate nutritional strategies into both medical practice and public health initiatives aimed at preventing vascular diseases.

The Mediterranean Diet Pyramid was first introduced in the *American Journal of Clinical Nutrition* in 1995, raising broad awareness about the MedDiet’s health benefits and cultural significance [2].

Traditionally, this eating pattern has been a hallmark of Mediterranean regions—including Southern Europe, North Africa, and the East Mediterranean. Historically, the diet featured a moderate to high intake of fats, mostly sourced from plants [3]. In its early forms, the MedDiet involved minimal meat intake, with red meats such as beef, pork, and lamb reserved for special occasions. Foods like processed meats, butter, ice cream, and other full-fat dairy products were rarely consumed, with dairy mostly limited to moderate amounts of fermented foods like cheese and yogurt.

Although the MedDiet contains a notable amount of fat, it remains largely based on plant foods, with olive oil as the main source. The diet prioritizes generous servings of fresh, locally sourced vegetables, fruits, legumes, nuts, and mainly whole grains. Fish and seafood were consumed in moderate quantities, varying by proximity to coastal areas, and served as the primary animal protein source.

Extra-virgin olive oil (EVOO) and red wine served as the primary sources of fat and alcoholic beverages, respectively. Olive oil was generously used in salads and in cooked dishes containing vegetables and legumes, while red wine was customarily consumed in moderate quantities alongside meals. These components not only enhanced the flavor and nutritional profile of the diet but also contributed to its beneficial health effects.

EVOO and red wine are abundant in bioactive polyphenols—including hydroxytyrosol, tyrosol, oleocanthal, and resveratrol—which are thought to possess anti-inflammatory effects. Traditionally, the cardiovascular benefits of olive oil have been linked to its high levels of monounsaturated fatty acids (MUFA), particularly oleic acid. More recent research suggests that polyphenols, especially those prevalent in EVOO and largely absent from refined olive oils, may provide additional protective benefits.

EVOO is obtained by first-cold pressing ripe olives and retains a high concentration of antioxidants, including polyphenols, tocopherols, and phytosterols. In contrast, refined and common olive oils undergo physical and chemical processing that preserves the lipid fraction but significantly diminishes their bioactive compound content, thereby reducing their antioxidant, anti-inflammatory, and pleiotropic potential [4].

The Mediterranean diet has been researched for its effects on metabolomics and the gut microbiome, with major consequences for cardiometabolic health [5].

Cerebrovascular disease, which includes various conditions that impact blood flow to the brain, continues to rank among the main causes of death, disability, and healthcare costs globally. Stroke, the most common form, affects more than 12 million people each year and leads to considerable long-term disability and financial burden [6].

Most strokes are ischemic and can be traced to changeable lifestyle and metabolic risk factors. The INTERSTROKE study and follow-up meta-analyses found that about 90% of stroke risk is linked to ten modifiable factors, such as high blood pressure, smoking, diabetes, abnormal blood lipids, obesity, lack of physical exercise, poor diet, and excessive alcohol consumption [7].

Lifestyle changes like the DASH (Dietary Approaches to Stop Hypertension) and Mediterranean diet, regular exercise, quitting smoking, and managing weight have demonstrated strong effectiveness in reducing these risk factors. As a result, a comprehensive understanding of the Mediterranean diet and its use in clinical practice provides an integrated approach to raising awareness of vascular health among patients. The aim of this work is to provide the reader with a clear overview of the latest scientific literature on the subject, allowing them to reflect on how the Mediterranean diet as a whole represents a valuable tool in addition to drug therapy for patients with cerebrovascular disease.

## 2. Key Components of the Mediterranean Diet

The MedDiet is widely acknowledged as a cornerstone of preventive medicine due to its robust association with reduced incidence of cardiovascular disease, metabolic disorders, and overall mortality [8]. From a clinical perspective, one of the most compelling demonstrations of the MedDiet’s efficacy is provided by the PREDIMED trial, a large-scale, multicenter, randomized controlled study that showed a 30% relative risk reduction in major cardiovascular events among high-risk individuals following a MedDiet supplemented with EVOO or mixed nuts [9]. This evidence has established the MedDiet not only as a preventive strategy but also as a therapeutic tool in managing cardiometabolic risk factors such as hypertension, dyslipidemia, insulin resistance, and central obesity. At the molecular level, the health-promoting properties of the MedDiet derive from the synergistic interactions of its bioactive components, which exert anti-inflammatory, antioxidant, and anti-thrombotic effects.

**Figure 1:**
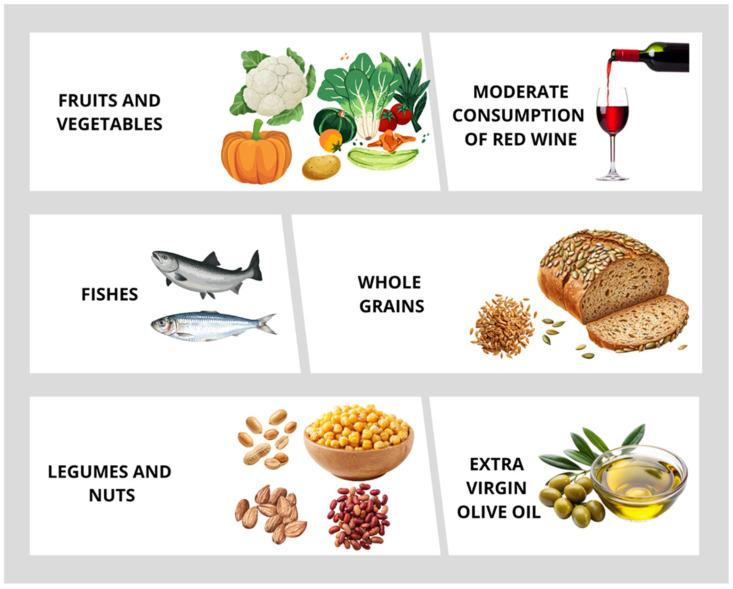
Main constituents of the Mediterranean diet.

The most important foods of the MedDiet, in detail, are the following (Figure 1).

- Extra-virgin olive oil (EVOO), which is especially high in mono-unsaturated fatty acids (primarily oleic acid) and phenolic compounds such as hydroxytyrosol and oleuropein; these molecules are known to modulate endothelial function, reduce oxidative stress, and lower chronic low-grade inflammation by blocking nuclear factor kappa-light-chain-enhancer of activated B cells (NF-κB) and activating nuclear factor erythroid 2-related factor 2 (Nrf2) signaling pathways; EVOO has also been found to improve lipid profiles by raising HDL cholesterol and lowering LDL oxidation, thereby decreasing the risk of atherosclerosis [10].
- Legumes and whole grains, another key part of the Mediterranean diet, provide fiber and resistant starches that can be fermented, boosting gut microbiota diversity and leading to the production of short-chain fatty acids (SCFAs) such as butyrate. These SCFAs help regulate immune function and strengthen the gut barrier, thereby improving insulin sensitivity and lowering systemic inflammation. Nuts are high in unsaturated fats, fiber, and polyphenols, and have been linked to lower LDL cholesterol, better endothelial function, and reduced inflammation markers, including C-reactive protein (CRP) and interleukin-6 (IL-6) [5].
- Fish, particularly oily varieties, is a primary source of long-chain omega-3 polyunsaturated fatty acids (eicosapentaenoic acid—EPA—and docosahexaenoic acid—DHA), which are incorporated into cell membranes and influence eicosanoid production, shifting it toward less inflammatory prostaglandins and leukotrienes. This mechanism is believed to explain the heart-protective and antiarrhythmic effects of omega-3 fatty acids [11].

Epidemiological studies show that adding polyphenol-rich fruits and vegetables to the diet helps prevent and reduce the consequences of aging on brain activity and behavior [12].

Further evidence from experimental animal research indicates that short-chain fatty acids produced by gut bacteria from dietary fiber can help suppress the development of various inflammatory, autoimmune, and allergic diseases [13]. In European Mediterranean regions, the diet often includes moderate wine consumption with meals. Some studies have aimed to identify biomarkers of polyphenol intake, especially those from specific food sources like red wine. Researchers have also found that polyphenols in the Mediterranean diet directly affect autophagy; for example, resveratrol—a polyphenol found in nuts, wine, and grapes—acts as an autophagy inducer [14].

The Mediterranean diet seems to protect vascular health not only through direct actions but also via indirect epigenetic changes. Recent nutrigenomic research has shown that following this diet alters gene expression patterns related to inflammation, lipid metabolism, and oxidative stress. These effects are partly due to changes in DNA methylation, histone alteration, and the control of non-coding RNAs [15]. This suggests that the benefits of the Mediterranean diet go beyond short-term metabolic improvements, potentially affecting disease risk throughout a person’s life.

In summary, the Mediterranean diet is a dietary model supported by science and relevant in clinical settings, offering multiple benefits. Its positive effects result not only from its healthy nutrient profile but also from complex interactions between dietary bioactives, host metabolism, immune responses, and gene regulation. These insights further support the Mediterranean diet’s role as a central approach for preventing and managing non-communicable diseases in both the general population and those at higher risk.

Randomized trials have underscored that the MedDiet confers superior cardiovascular protection compared with other dietary patterns, including low-fat and low-carbohydrate regimens. In the PREDIMED trial, participants at high cardiovascular risk randomized to a MedDiet enriched with EVOO or mixed nuts experienced a 30% relative risk reduction in major adverse cardiovascular events (MACE) compared to those assigned to a low-fat control diet [9]. MedDiet’s high content of monounsaturated and polyunsaturated fats (predominantly oleic and linoleic acids) promotes favorable lipid remodeling—elevating HDL cholesterol, reducing LDL oxidation, and attenuating triglyceride levels—whereas low-fat diets often diminish beneficial fatty acid intake, potentially undermining these lipid-modulatory effects [16,17].

In the CORDIOPREV trial, which assigned more than 1000 patients with coronary artery disease to either a Mediterranean or a low-fat diet, long-term adherence to the MedDiet was linked to a significant reduction in carotid intima-media thickness and slowed plaque growth (HR 0.719; 95% CI 0.541–0.957). In comparison, the low-fat diet group showed smaller improvements in vascular structure [18]. The Mediterranean diet also promotes beneficial changes in gut microbiota and increases the production of short-chain fatty acids (such as butyrate), which help lower systemic inflammation—an effect only partly observed with high-fiber DASH diets. These changes improve insulin sensitivity and reduce C-reactive protein levels [19].

By contrast, low-carbohydrate diets can lead to quick weight loss and short-term improvements in blood sugar control, but they often involve higher consumption of saturated fats and less intake of fiber-rich plant foods. This limits their ability to protect heart health over the long term [17].

Vegetarian and vegan diets, while associated with reductions in LDL cholesterol, may not inherently ensure adequate intake of bioactive lipids and polyphenols characteristic of MedDiet, leading to heterogeneity in vascular outcomes unless meticulously balanced [20].

In summary, the Mediterranean diet’s combination of heart-protective fats, antioxidants, anti-inflammatory plant compounds, and fibers that support gut health explains its consistently stronger effects on both clinical outcomes and vascular health markers compared to other diets. This evidence supports recommending the Mediterranean diet for primary and secondary prevention of cardiovascular events.

## 3. Protective Mechanisms of the Mediterranean Diet

### 3.1. Anti-Inflammatory and Antioxidant Effects

The Mediterranean diet is well known for its antioxidant and anti-inflammatory features, which help protect against chronic degenerative diseases [21]. The Mediterranean diet provides abundant vitamins and polyphenols, especially from fruits, vegetables, whole grains, nuts, extra-virgin olive oil, and red wine. Key dietary antioxidants include beta-carotene (a precursor of vitamin A), vitamin C, vitamin E, and, sometimes, the trace element selenium. Most polyphenols exhibit low oral bioavailability due to poor absorption in the upper gastrointestinal tract, extensive metabolism (phase I/II hepatic and intestinal), and transformation by gut microbiota, resulting in circulating metabolites that differ substantially from the parent compounds studied in vitro [22]. In vitro studies typically use native polyphenols at concentrations not achievable in vivo, neglecting the fact that only a small fraction of the ingested dose reaches systemic circulation as the parent compound; instead, bioactive effects in vivo are often mediated by metabolites produced during digestion and microbial catabolism [23]. Interindividual variability in bioavailability is pronounced, driven by differences in gut microbiota composition, genetic polymorphisms, age, sex, and dietary factors, further complicating extrapolation from in vitro to in vivo contexts [24]. 

Olive oil, a main element of the Mediterranean diet, is rich in polyphenols that help lower the risk of metabolic syndrome. These polyphenols block NF-κB activation and production, a key regulator in the development of metabolic syndrome, thereby lowering the production of proinflammatory cytokines [5]. Plants produce polyphenols in response to environmental challenges, such as radiation or pathogen attacks, and these protective effects are believed to also benefit human health. Because they contain several hydroxyl groups, polyphenols act as strong antioxidants and exhibit significant anti-inflammatory properties [25]. In particular, they can influence important proinflammatory signaling pathways, including the NLR family pyrin domain containing 3 (NLRP3) inflammasome, NF-κB, and mitogen-activated protein kinase (MAPK). Lab studies have shown that polyphenols may inhibit these pathways, reducing the production and release of proinflammatory cytokines such as interleukins (IL-1β, IL-6, IL-8) and tumor necrosis factor-alpha (TNF-α), all of which are linked to adverse cardiovascular outcomes. However, despite their potent anti-inflammatory effects observed in vitro, the translation of these benefits in vivo remains uncertain, largely due to the considerable variability in polyphenol bioavailability.

**Figure 2:**
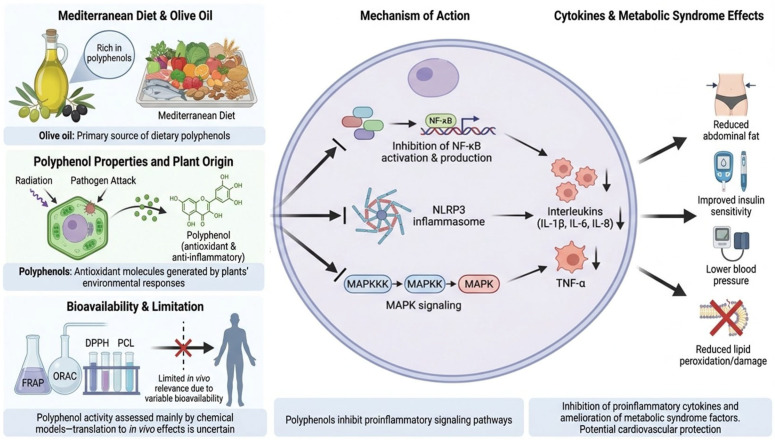
Effects of olive oil-derived polyphenols on metabolic syndrome and inflammatory pathways (NF-κB: Nuclear factor kappa-light-chain-enhancer of activated B cells; IL-1β: interleukin-1 beta; NLRP3: NLR family pyrin domain containing 3 inflammasome; MAPK: Mitogen-Activated Protein Kinase; MAPKK: Mitogen-Activated Protein Kinase Kinase; MAPKKK: Mitogen-Activated Protein Kinase Kinase Kinase; IL-6: Interleukin-6; IL-8: Interleukin-8; TNF-α: Tumor Necrosis Factor alpha; DPPH: 2,2-Diphenyl-1-picrylhydrazyl; PCL: Photochemiluminescence; FRAP: Ferric Reducing Antioxidant Power; ORAC: Oxygen Radical Absorbance Capacity, ↓: reduction in concentration).

Polyphenols’ antioxidant properties are mostly measured with chemical models and plant extracts using various laboratory techniques. Commonly used methods include ferric reducing antioxidant power (FRAP), oxygen radical absorbance capacity (ORAC), 2,2-diphenyl-1-picrylhydrazyl (DPPH), and photochemiluminescence (PCL), which are useful for quickly evaluating antioxidant activity. However, whether used alone or together, these techniques do not truly mimic the complexity of the human body, so their real-world relevance is limited (Figure 2) [26].

A further distinctive aspect of the Mediterranean diet is its moderate red wine consumption, with resveratrol as the main polyphenol, known for its strong antioxidant and anti-inflammatory effects. Resveratrol has also been associated with positive changes in gut microbiota, a key factor for metabolic health, and with the activation of sirtuin 1, which promotes lipolysis, and adenosine monophosphate-activated protein kinase (AMPK), which enhances insulin sensitivity; it was also shown to inhibit NLRP3 activation by triggering autophagy and preserving mitochondrial function in both cell and animal studies. Additionally, resveratrol reduced liver inflammation in mice with diet-induced obesity [27].

In the last five years, many laboratory and animal studies have shown that dietary phenolic compounds found in several Mediterranean diet foods have anti-inflammatory effects by modulating the inflammasome. This process works through several mechanisms. First, high levels of mitochondrial reactive oxygen species (ROS) promote activation of the inflammasome, a key molecular platform linking oxidative stress to inflammation. This activation then stimulates caspase-1, leading to the maturation and release of interleukin-1β (IL-1β) [28]. This chain reaction initiates a broader inflammatory cascade involving the Toll-like receptor (TLR) family, especially TLR1 and TLR2, which are vital to the innate immune system and are also expressed in organs such as the liver and gastrointestinal tract.

Other compounds with antioxidant properties can inhibit key molecular pathways involved in systemic inflammation. For example, Hori et al. found that a standardized Brazilian green propolis extract (EPP-AF)—rich in cinnamic acid derivatives such as p-coumaric acid—reduced IL-1β secretion and lowered caspase-1 activation in mouse macrophages [29]. Similarly, flavonoids like apigenin and procyanidin B2 have been shown to block inflammasome activation and IL-1β production in LPS-stimulated human macrophage models: apigenin interfered with NLRP3 inflammasome assembly and suppressed caspase-1 activation in macrophages derived from human monocytic leukemia (THP-1-derived macrophages) [30], while procyanidin B2 reduced inflammasome-mediated IL-1β production in Lipopolysaccharide-primed THP-1 macrophages [31]. Recent reviews confirm that polyphenols can dampen inflammasome activation mainly by decreasing oxidative stress/ROS and mitochondrial dysfunction [32]. Since NLRP3 activation is strongly associated with metabolic inflammatory diseases, including type 2 diabetes, these findings highlight the therapeutic potential of dietary polyphenols in reducing chronic inflammation [33,34].

### 3.2. Modulation of Lipid and Glucose Metabolism

The International Diabetes Federation (IDF) estimates that the global prevalence of diabetes will reach 700 million individuals by 2045 [35]. The Mediterranean diet’s effectiveness in lowering glycated haemoglobin (HbA1c) is well supported, though the size of its effect varies depending on the comparison diet [36]. Network meta-analyses indicate that no single diet is always superior in every comparison, and the Mediterranean diet may not outperform some alternatives (such as the Paleolithic diet) for HbA1c based on limited head-to-head data [37]. Notably, in people with newly diagnosed type 2 diabetes, a randomized trial and long-term follow-up showed better blood sugar outcomes and a delayed need for glucose-lowering medications with a low-carbohydrate Mediterranean-style diet compared to a low-fat diet [38].

The Mediterranean diet has been shown to enhance Glucagon-like peptide-1 (GLP-1) activity: it is an incretin hormone that plays an important role in regulating glucose balance by promoting insulin secretion when glucose is present, slowing gastric emptying, and reducing glucagon release—thus supporting effective management of T2D [39,40]. Beyond blood sugar regulation, it also improves endothelial function under oxidative stress by triggering antioxidant defenses in the endothelium (such as antioxidant enzymes HO-1 and NQO1) [41]. However, persistent high blood sugar is linked to increased oxidative and endoplasmic reticulum (ER) stress and worsening β-cell function; mechanistically, ER stress in pancreatic β-cells can lead to “incretin desensitization” (a kind of GLP-1 resistance) and further β-cell dysfunction [42], as part of the broader effect of oxidative stress on β-cells in type 2 diabetes.

The MedDiet represents a potential dietary approach to counteract this dysfunction. In patients with type 2 diabetes, a Mediterranean-style meal has been shown to increase postprandial incretin levels compared to a vegetarian meal [43], and a single serving of extra-virgin olive oil raised postprandial GLP-1 more than other tested oils in a controlled study [44]. Mechanistically, long-chain unsaturated fatty acids—found in olive oil and other foods—can activate fatty-acid-sensing G protein-coupled receptors (GPCRs), such as Free Fatty acid receptor 4 (FFAR4), also termed G-protein coupled receptor 120 (GPR120) [FFAR4/GPR120], which are involved in stimulating incretin secretion from enteroendocrine L-cells [45]. Finally, clinical evidence suggests that a Mediterranean diet with added olive oil can improve markers of oxidative stress and inflammation and enhance endothelial responsiveness to GLP-1 during hyperglycemia [46].

Atherosclerosis is the main underlying factor for myocardial infarction (MI) in over 90% of cases, while large-artery atherosclerosis accounts for a smaller share of ischemic strokes (about 25%) [47,48]. Population-based prospective studies consistently show that reducing saturated fat intake reduces coronary heart disease (CHD) risk when it is replaced with unsaturated fats or high-quality carbohydrates. In a large pooled analysis, substituting 5% of energy from saturated fat with polyunsaturated fat, monounsaturated fat, or whole-grain carbohydrates reduced the risk of CHD by 25%, 15%, and 9%, respectively [49].

On the other hand, replacing saturated fats with refined starches or added sugars did not significantly affect CHD risk in that analysis. Long-term randomized dietary trials also confirm cardiovascular benefits: a Cochrane meta-analysis found that reducing saturated fat for at least 24 months lowered combined cardiovascular events by 17% (RR 0.83; 95% CI 0.70–0.98) [50]. However, effect estimates differ among meta-analyses depending on included studies and methods (for example, one Nutrition Journal meta-analysis found no significant effects on some outcomes) [51]. Notably, genetic studies show that earlier and prolonged exposure to lower LDL levels provides greater protection than starting LDL reduction later in life [52]. Proprotein convertase subtilisin/kexin type 9 (PCSK9) loss-of-function variants, which result in lifelong lower LDL, are associated with much lower CHD risk (about 28% lower LDL with 88% less CHD risk in Black participants, and about 15% lower LDL with 47% less CHD risk in White participants) [53]. Similarly, people with inactivating 13-transmembrane domain cell surface cholesterol-sensing receptor (NPC1L1) mutations had about 12 mg/dL lower LDL and 53% lower CHD risk (OR 0.47; 95% CI 0.25–0.87). Nuts (such as walnuts, almonds, pistachios, and hazelnuts) have also demonstrated cholesterol-lowering effects in clinical trials and meta-analyses [54,55,56].

Prospective cohort studies show that people who regularly eat nuts have a lower risk of coronary heart disease. For example, in the Nurses’ Health Study, women who ate more than five one-ounce servings per week had a 35% lower risk of total CHD (RR 0.65) and a similar reduction in fatal CHD (RR 0.61) [57]. In the Physicians’ Health Study, men who ate nuts at least twice a week had a 47% lower risk of sudden cardiac death (RR 0.53) and a 30% lower risk of total CHD death (RR 0.70) [58]. In a six-month randomized controlled trial, a cholesterol-lowering “dietary portfolio” that included plant sterols, soy protein, viscous fibers, and nuts lowered LDL-C by 13.8% (intensive counseling) and 13.1% (routine counseling), compared to just 3.0% with a low–saturated fat control diet [59]. Tree nuts have also been found to improve lipid levels in controlled feeding studies: a meta-analysis of 61 intervention trials estimated that eating one 1-oz serving per day lowers LDL-C by 4.8 mg/dL and total cholesterol by 4.7 mg/dL [60].

Other mechanisms also help explain how the Mediterranean diet lowers LDL cholesterol. One review notes that the Mediterranean diet supplies at least 14 g of fiber per 1000 kcal, with fermentable fibers supporting the formation of short-chain fatty acids (SCFAs) and providing overall metabolic benefits [61].

### 3.3. Impact on Endothelial Function and Vascular Health

**Figure 3:**
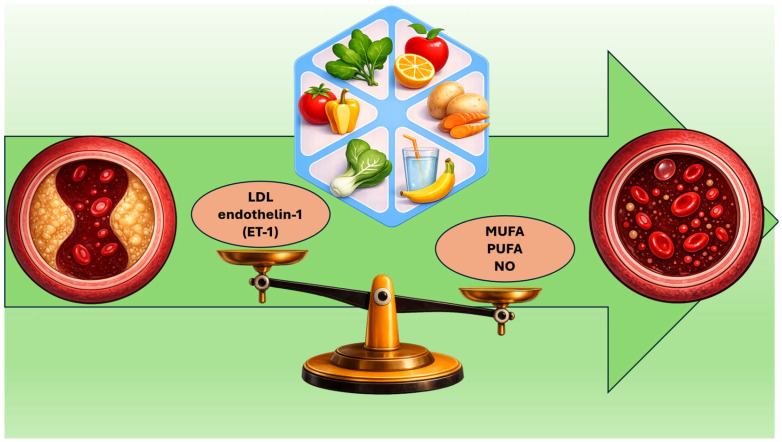
Role of the Mediterranean diet in restoring vascular health through modulation of the balance between LDL/endothelin-1 on one side and nitric oxide and mono-unsaturated/poly-unsaturated fatty acids on the other, thereby improving endothelial function and blood flow (LDL: Low-Density Lipoprotein, NO: Nitric Oxide, MUFA: Mono-Unsaturated Fatty Acids, PUFAs: Poly-Unsaturated Fatty Acids).

The Mediterranean diet has a major impact on endothelial function and vascular health through its lipid-lowering, anti-inflammatory, and antioxidant actions (Figure 3). Long-term randomized studies show that reducing saturated fat intake—especially when replaced by unsaturated fats or complex carbohydrates—lowers the risk of cardiovascular events [50]. On the vascular level, the unsaturated fats in the Mediterranean diet (such as Poly-Unsaturated Fatty Acids—PUFAs—from nuts and Monounsaturated fatty acid—MUFAs—from extra-virgin olive oil) and the phenolic compounds in EVOO are linked to better endothelial reactivity (for example, improved reactive hyperaemia index—RHI), which reflects enhanced nitric oxide–mediated vasodilation [62,63,64]. Fermentation of fiber by gut microbiota produces short-chain fatty acids (SCFAs), such as butyrate and propionate. These act as signaling and epigenetic molecules (including histone deacetylase inhibition) and can affect intestinal cholesterol absorption (for example, through effects on NPC1L1-mediated absorption as seen with propionate) [65].

**Figure 4:**
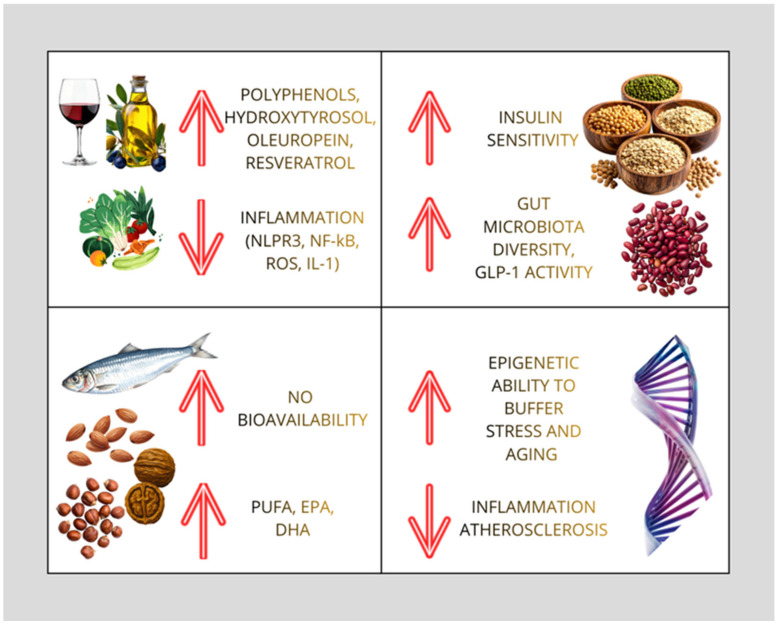
Pleiotropic effects of the Mediterranean diet on multiple biological pathways, including molecular and epigenetic mechanisms (GLP-1: Glucagon-like peptide-1; NLRP3: NLR family pyrin domain containing 3 inflammasome; NF-κB: nuclear factor kappa-light-chain-enhancer of activated B cells; ROS: reactive oxygen species; IL-1: interleukin-1; NO: nitric oxide; PUFA: Poly-Unsaturated Fatty Acids; EPA: eicosapentaenoic acid; DHA: docosahexaenoic acid; ↑: increase; ↓: reduction).

Phytosterols, found in nuts, seeds, and unrefined vegetable oils, inhibit intestinal cholesterol absorption by displacing cholesterol from mixed micelles, and supplementation can significantly lower LDL cholesterol in people with high cholesterol, as shown in meta-analyses [66]. Beyond their effects in the intestine, phytosterols can be incorporated into cell membranes and change their biophysical properties in lab models [67]. Because cholesterol-rich membrane microdomains (“lipid rafts”) are important for receptor signaling and vascular inflammation, changes in membrane sterol composition are biologically relevant, even though their effects on blood vessels in humans are still being studied [68]. In addition, polyphenols—especially those in extra-virgin olive oil (such as hydroxytyrosol) and red wine (such as resveratrol)—support endothelial health by boosting the production of antioxidant enzymes (like catalase and superoxide dismutase), reducing the generation of ROS by NADPH oxidase (NOX), and preserving nitric oxide (NO) availability (Figure 4) [69,70].

## 4. Mediterranean Diet and Cerebrovascular Risk Factors

The MedDiet, abundant in fruits, vegetables, whole grains, legumes, nuts, olive oil, and includes a moderate amount of fish and wine, provides strong protection against a broad range of cerebrovascular risk factors. These include hypertension, dyslipidaemia, atherosclerosis, insulin resistance, and type 2 diabetes, all of which are major modifiable contributors to stroke and cognitive decline.

### 4.1. Mediterranean Diet and Diabetes

Although type 2 diabetes (T2D) is influenced by both genetic and environmental factors, healthy lifestyle choices—especially diet—are essential for its prevention. Interventions based on the Mediterranean diet can help reduce central or visceral fat, which is a major contributor to insulin resistance. In the PREDIMED-Plus randomized clinical trial (interim subgroup analysis), combining an energy-reduced Mediterranean diet with physical activity led to significant reductions in total and visceral fat compared to advice to follow a regular Mediterranean diet [71]. Mediterranean-style diets have also been linked to notable decreases in waist circumference in randomized trials. Regarding antioxidant micronutrients, a randomized trial in adults with T2D tested high-dose vitamin A (50,000 IU) plus vitamin E (100 mg), with or without zinc (25 mg), over three months; compared to control, the vitamin A+E+zinc group had reductions in fasting blood glucose and HbA1c, although the authors emphasized the need for caution with vitamin A doses [72]. Vitamin A may also affect T2D biology through mechanisms related to redox balance and gene regulation, particularly when deficient [73]. Further prospective evidence supports a link between greater adherence to the Mediterranean diet and lower incidence of T2D. Earlier systematic reviews and meta-analyses reported a 19% lower risk of diabetes for those with the highest compared to the lowest Mediterranean diet adherence [74], while a recent updated dose–response meta-analysis of prospective cohort studies (through January 2022) found a pooled relative risk of 0.83 for the highest versus lowest adherence [75]. In people with prediabetes, a Spanish primary-care cohort study found that high adherence to the MediDiet was associated with a lower progression to T2D [76].

Results from the PREDIMED-Reus randomized trial (with 418 nondiabetic adults aged 55–80 years on ad libitum diets and no set physical activity) showed that a Mediterranean diet with extra-virgin olive oil (EVOO) or mixed nuts reduced new cases of type 2 diabetes compared to advice for a low-fat diet; when both Mediterranean diet groups were combined, the incidence of diabetes dropped by 52% [77]. Consistent results were found in a multicenter PREDIMED subgroup analysis of participants without diabetes at baseline (number = 3541): compared to the low-fat advice group, the MedDiet+EVOO group had a significantly lower risk of developing diabetes (multivariable-adjusted HR 0.60), while the MedDiet+nuts group did not reach statistical significance (HR 0.82) [78].

Concerning prospective studies, a systematic review and meta-analysis found that greater adherence to several “heart-healthy” dietary patterns—including the Mediterranean diet (RR 0.87) and DASH (RR 0.81)—was associated with significantly fewer new cases of diabetes [79]. Another systematic review and meta-analysis focused on Mediterranean diets without fat restriction also concluded that this eating pattern may reduce the risk of T2D [80].

The DASH dietary pattern is commonly implemented with sodium targets of about 1500 to 2300 mg per day. Notably, vegetarian or plant-based diets are also linked to a lower risk of abnormal blood sugar: in the Rotterdam Study, a higher plant-based dietary index (for each 10-point increase) was linked to lower prediabetes risk (HR 0.89) and lower type 2 diabetes risk (HR 0.82), though the association weakened after adjusting for BMI [81]. More recently, an updated meta-analysis of prospective studies confirmed that people who more closely follow plant-based diets—especially “healthy” versions with whole grains, legumes, fruits, and vegetables—have a lower risk of developing type 2 diabetes [82].

Vegetarian dietary patterns focus on whole plant foods while limiting animal products to varying degrees: vegan diets exclude all animal products, lacto-ovo-vegetarian diets allow dairy and eggs but no meat, pescatarian diets include fish, and semi-vegetarian diets allow limited meat intake. Evidence shows that higher meat consumption—especially processed and unprocessed red meat—is associated with a greater risk of type 2 diabetes. Therefore, vegetarian patterns may offer metabolic benefits by replacing meat with plant-based foods.

Regarding the Mediterranean diet, the ATTICA study found that among participants with normal blood sugar, those in the highest third of adherence had 7% lower fasting glucose, 5% lower fasting insulin, and 15% lower Homeostatic Model Assessment of Insulin Resistance (HOMA-IR) than those in the lowest third. Among participants with diabetes or impaired fasting glucose, the highest-adherence group showed 15% lower glucose, 15% lower insulin, and 27% lower HOMA-IR, but these associations were confirmed by multivariable analysis only among normoglycemic individuals [83].

Finally, in a randomized PREDIMED substudy (n = 772; 3-month follow-up), the Mediterranean diet supplemented with virgin olive oil and the diet supplemented with nuts led to mean reductions in plasma glucose of −0.39 mmol/L (95% CI −0.70 to −0.07) and −0.30 mmol/L (95% CI −0.58 to −0.01), respectively, compared to a low-fat diet [84].

### 4.2. Mediterranean Diet and Hypertension

Regarding blood pressure, a systematic review and meta-analysis of randomized trials (six studies, over 7000 participants, interventions lasting at least one year) found that MedDiet interventions reduced systolic blood pressure by 1.44 mmHg and diastolic blood pressure by 0.70 mmHg. The authors noted, however, that these findings should be considered cautiously due to the reduced number of eligible trials [85]. More recent evidence is consistent with these modest effects: a 2021 systematic review and meta-analysis of 19 RCTs (n = 4137) found that the Mediterranean diet lowered systolic blood pressure by 1.4 mmHg and diastolic blood pressure by 1.5 mmHg versus control [86]. In another 2021 meta-analysis of 35 RCTs (n = 13,943), the MedDiet reduced systolic blood pressure (SBP) by at least 1.5 mmHg and diastolic blood pressure (DBP) by 0.9 mmHg compared with usual diets or other interventions; the reductions were greater with longer follow-up and higher initial systolic blood pressure [87].

In an Australian randomized controlled trial (MedLey; 166 adults aged over 64 years), participants assigned to follow a Mediterranean diet for six months experienced small but significant reductions in SBP and improved endothelial function; flow-mediated dilation increased by 1.3% after six months [88].

Findings from the PREDIMED trial also support the blood pressure benefits of the Mediterranean diet: over time, the Mediterranean diet supplemented with extra-virgin olive oil or nuts was linked to lower diastolic blood pressure compared to the low-fat control diet [89]. In a PREDIMED substudy using ambulatory monitoring after one year, the Mediterranean diet groups (EVOO or nuts) had reductions in 24 h ambulatory systolic blood pressure, and similar reductions were seen for diastolic blood pressure [90].

Observational data from Italy support these findings: in a recent work of our group, we demonstrated that lower adhesion to a Mediterranean-style diet is linked to a worse profile of cardiovascular risk factors in patients with hypertension; therefore, following a Mediterranean diet in individuals with hypertension is related to a better cardiometabolic profile [91].

### 4.3. Mediterranean Diet and Lipid Levels

Metabolic syndrome (MetS) is linked to atherogenic changes in the structure and function of lipoproteins, such as increased triglycerides, more small and dense LDL, and shifts in HDL quality. These changes can be measured using advanced lipoprotein testing (ADLT), often with nuclear magnetic resonance (NMR) profiling [92]. Atherosclerotic cardiovascular disease is still a major global cause of illness and death. Mechanistically, retention and oxidation of apoB-containing lipoproteins in the artery wall trigger endothelial activation and attract leukocytes; the monocytes that enter turn into macrophages, which take up modified LDL (using scavenger receptor pathways), forming foam cells that fuel plaque growth and vascular inflammation [93].

A 2024 randomized controlled sub-study within PREDIMED-Plus by Candás-Estébanez et al. compared a regular Mediterranean diet with an energy-restricted Mediterranean diet plus physical activity (er-MedDiet + PA) in patients with metabolic syndrome. The results showed that, in addition to improvements in conventional lipid levels, advanced lipoprotein testing (ADLT) detected a decrease in small dense LDL-cholesterol (sd-LDL-C), intermediate-density lipoproteins, VLDL-triglyceride, and HDL-triglyceride, as well as an increase in large LDL and large VLDL particles in the er-MedDiet + PA group after 12 months [94].

Therapeutic strategies are increasingly incorporating lifestyle-based approaches and bioactive compounds typical of the Mediterranean diet. Extra-virgin olive oil (EVOO) provides monounsaturated fatty acids and a rich phenolic content with proven antioxidant and anti-inflammatory effects; Mediterranean dietary patterns that emphasize olive oil have been discussed as modulators of vascular and cardiometabolic pathways, including via nutrigenetic and epigenetic mechanisms. Additionally, Mediterranean foods such as olive oil, wine, nuts, herbs, and spices provide polyphenols and other secondary metabolites; strategies to improve the delivery and bioavailability of these compounds have been reviewed in the context of the Mediterranean diet [95].

Preclinical research on omega-3 polyunsaturated fatty acids supports their mitochondrial and neurobiological benefits. Afshordel et al. found that administering fish oil orally for 21 days in young and aged mice increased omega-3 PUFA derivatives and improved mitochondrial function and ATP-related outcomes in brain tissue [96].

Long-term adherence to a Mediterranean diet is consistently linked to a lower burden of subclinical atherosclerosis and slower progression of vascular disease. In the CORDIOPREV randomized controlled trial (for secondary prevention), a Mediterranean diet high in extra-virgin olive oil (EVOO) lowered common carotid intima–media thickness and reduced carotid plaque height over 5–7 years compared with a low-fat diet [18]. Similarly, in the ILERVAS prospective cohort, higher adherence to the Mediterranean diet predicted lower incidence and progression of subclinical atherosclerosis, while lower adherence was linked to more frequent and numerous atherosclerotic plaques [97]. From a public health standpoint, leading scientific societies highlight the importance of improving overall dietary patterns—rather than focusing on single nutrients—as a primary strategy for reducing atherosclerotic cardiovascular disease risk.

Extra-virgin olive oil (EVOO) also supplies minor bioactive compounds, such as phytosterols. Laboratory studies show that some phytosterols can selectively inhibit cyclooxygenase-2 (COX-2) activity [98], suggesting a possible additional anti-inflammatory effect alongside their well-known cholesterol-lowering action, which occurs by competing with cholesterol for intestinal absorption.

Carotenoids (such as α-carotene, β-carotene, lycopene, lutein, zeaxanthin, β-cryptoxanthin, and astaxanthin) are abundant in plant foods that are central to the Mediterranean diet and possess antioxidant and immunomodulatory properties [99]. In animal studies, β-carotene supplementation was found to speed up the resolution of atherosclerosis, supporting a mechanistic link between carotenoids and plaque dynamics [100]. Consistent with the food-matrix effect, a randomized, crossover feeding trial found that tomato sauce enriched with olive oil led to greater improvements in certain cardiovascular biomarkers than raw tomato or plain tomato sauce [101]. A systematic review and meta-analysis of data from intervention trials found that tomato products and/or lycopene have beneficial effects on several cardiovascular risk factors, including lipid-related factors [102]. Finally, nuts—a staple of the Mediterranean diet—are among the richest dietary sources of coenzyme Q10, along with meat, fish, and certain oils [103].

## 5. Mediterranean Diet and Ischemic Stroke

Ischemic stroke, the most common subtype, is strongly associated with modifiable lifestyle factors, particularly diet.

Further analyses within the broader PREDIMED framework confirm that the benefits of the Mediterranean diet are consistent across different levels of baseline cardiovascular risk. For instance, a post hoc analysis using Framingham-REGICOR risk categories found that adherence to or intervention with the Mediterranean diet was associated with a reduced risk of MACE, with no significant difference across initial risk categories (major CVD endpoints included myocardial infarction, stroke, and cardiovascular death) [104].

Mechanistically, the Mediterranean diet is believed to lower the risk of ischemic stroke through several converging pathways, including favorable effects on systemic inflammation, endothelial function and nitric oxide availability, platelet activation, blood pressure, insulin sensitivity, and the burden of atherosclerotic risk factors. These mechanisms align with broader evidence supporting heart-healthy dietary patterns, including Mediterranean-style diets, for cardiovascular prevention [105]. In recent years, reviews of epidemiological data have further strengthened the link between greater adherence to the Mediterranean diet and reduced stroke risk. A systematic review of prospective studies found an inverse association between MedDiet adherence and stroke risk [106], and more recent reviews have continued to support this protective association [107].

Beyond prevention, the evidence for the Mediterranean diet’s impact on post-stroke functional recovery is still limited, but observational studies suggest a prognostic benefit. Among patients with acute ischemic stroke subjected to endovascular thrombectomy, those with greater adherence to a Mediterranean dietary pattern were more likely to achieve a favorable 3-month functional outcome (mRS 0–2) [108]. More recently, another observational study found that higher adherence to the Mediterranean diet before stroke was linked to early neurological improvement and a better 3-month prognosis after ischemic stroke [109]. However, direct interventional evidence on the implementation of the Mediterranean diet after stroke remains limited; a systematic review of dietary trials in stroke survivors reported possible improvements in cardiometabolic risk factors but stressed the need for larger, well-designed studies focused on clinical outcomes [110].

Mechanistically, bioactives in the Mediterranean diet may help support recovery by targeting neuroinflammation, oxidative stress, endothelial dysfunction, and neurovascular repair. In experimental models of ischemic stroke, long-term dietary supplementation with the plant-derived omega-3 fatty acid alpha-linolenic acid (ALA) reduced infarct size, improved neurological outcomes, and protected blood–brain barrier integrity, while also reducing inflammatory activation [111]. In models where brain omega-3 levels are chronically elevated (such as fat-1 transgenic mice), omega-3 enrichment has been shown to boost endogenous neurogenesis after focal cerebral ischemia [112]. As for polyphenols, recent stroke-focused reviews suggest that these compounds can target several aspects of the ischemic cascade—including oxidative/nitrative stress, inflammatory signaling, apoptosis, and excitotoxicity—supporting a biologically plausible neuroprotective role [113].

Beyond the approximately 30% reduction in major cardiovascular events seen in the updated PREDIMED primary-prevention analysis [114], subsequent PREDIMED and PREDIMED-Plus ancillary studies have identified additional pathways that may be relevant to stroke:

- micronutrient status (iron): In a nested case–control study within PREDIMED, higher serum iron levels were inversely associated with the odds of a first cardiovascular event. When checking the highest to the lowest quartile, the multivariable-adjusted odds ratio was 0.55 (95% CI 0.32–0.93; *p*-trend = 0.020), and the association was even stronger among women (OR 0.15; 95% CI 0.03–0.69; *p*-trend = 0.011) [115];
- cellular aging: In PREDIMED-Plus, a three-year lifestyle intervention using an energy-reduced Mediterranean diet plus physical activity did not affect telomere length in the overall cohort, but there was a significant difference by sex; women in the intervention group experienced an increase in telomere length and a lower risk of telomere shortening (OR 0.17; 95% CI 0.05–0.64) [116];
- diet quality and ultra-processed foods: in a cross-sectional baseline analysis from PREDIMED-Plus (n = 645), higher intake of ultra-processed meals (as a percentage of total energy) was linked to lower adherence to the Mediterranean diet and differences in certain gut microbiota taxa [117];
- body composition imaging: In an interim subgroup analysis of the PREDIMED-Plus randomized trial (DXA subsample n = 1521), a 3-year intervention using an energy-reduced Mediterranean diet plus physical activity reduced both total and visceral fat and slowed the loss of lean mass compared to advice to follow an unrestricted Mediterranean diet [71];
- cardio-embolic substrate and alcohol: in 503 PREDIMED-Plus participants with serial echocardiography, alcohol intake (per 1 drink per day) was associated with a larger left atrial volume index and lower left atrial reservoir and contractile strain in cross-sectional analysis; increases in alcohol consumption over time were linked to left atrial enlargement during follow-up [118];
- inflammatory profile and lifestyle: in a prospective analysis from PREDIMED-Plus (n = 489), increases in total, moderate, and moderate-to-vigorous physical activity over one year were linked to a decrease in a composite inflammatory score, which included IL-6, IL-8, IL-18, MCP-1, C-peptide, hs-CRP, leptin, and RANTES [119];

Despite strong evidence supporting the Mediterranean diet (MedDiet) in reducing cerebrovascular risk, several methodological gaps and limitations warrant further consideration. First, comparability between dietary patterns (e.g., MedDiet vs. DASH) is limited by substantial heterogeneity in operative definitions and adherence scores, which may capture non-equivalent food substitutes and bioactive profiles. This contributes to incongruent results across studies and settings, particularly when considering hard endpoints such as stroke or major adverse cardiovascular events. Secondly, even within randomized controlled trials, part of the observed benefit may reflect the intensity of dietary counseling and participant adherence, while in PREDIMED, supplementation with extra virgin olive oil or nuts complicates the attribution of effects to the overall dietary pattern rather than to specific components. Furthermore, the literature is considerably more robust for the primary and secondary prevention of cardiovascular events than for neurological endpoints after stroke: evidence on functional recovery and post-stroke rehabilitation remains largely observational, and systematic reviews highlight the need for larger, well-designed intervention studies focusing on neurological clinical outcomes.

Finally, preclinical studies on neuroprotection provide biological plausibility. In a male rat model of transient middle cerebral artery occlusion (tMCAO), a four-week pre-stroke Mediterranean-like diet enriched with hydroxytyrosol (with or without exercise) was linked to smaller infarct and edema volumes during follow-up (the diet group showed the greatest downward trend, although the difference in infarct volume did not have statistical significance at the specified time point; *p* = 0.1717 at day 14). This was accompanied by a lower systemic inflammatory profile (reduced baseline levels of several cytokines and chemokines, including IL-6), less oxidative DNA damage as measured by 8-OHdG, and better short-term functional recovery [120].

In summary, the growing body of evidence from PREDIMED and PREDIMED-Plus connects Mediterranean diet intake to measurable improvements in iron status, telomere dynamics (as a marker of biological aging), diet quality and gut microbiota features related to ultra-processed food consumption, body fat distribution (less visceral fat, better lean mass preservation), heart structure and function signals relevant to cardioembolic risk, and lower systemic inflammation (Table 1). Experimental studies also show that olive-derived phenolics can downregulate inflammasome pathways. Along with the reduction in clinical events observed in PREDIMED, these findings support a link between dietary habits and molecular signaling and vascular/brain resilience, aligning with protection against ischemic stroke.

## 6. Post-Stroke Mediterranean Diet Adherence

After an acute ischemic stroke (AIS), adopting—and sustaining—high adherence to a Mediterranean dietary pattern represents a pragmatic cornerstone of lifestyle-based secondary prevention, because it targets several mechanistic “drivers” of recurrence (blood pressure, atherogenic dyslipidemia, insulin resistance, adiposity, endothelial dysfunction, and low-grade inflammation) while remaining feasible within real-world rehabilitation pathways. Contemporary secondary-prevention guidance from the American Heart Association/American Stroke Association explicitly recommends low-salt and/or Mediterranean-type dietary patterns as part of comprehensive risk reduction in patients with prior stroke or transient ischemic attack, positioning diet alongside pharmacological strategies and structured physical activity [123].

Yet, in the post-stroke setting, adherence is neither automatic nor uniformly achievable: stroke-related disability can directly impair the “behaviors” that operationalize diet quality (shopping, cooking, meal planning), and qualitative work in chronic community-dwelling survivors highlights how fatigue, hemiparesis, perceptual deficits, and negative affect can translate into reduced autonomy, constrained food choice, and diminished social participation around meals—factors that plausibly erode adherence over time if not proactively addressed [124].

In parallel, observational evidence indicates that adherence can be socially patterned; for instance, in a cohort of patients with cardiovascular-related conditions (including stroke), material and social deprivation emerged as key correlates of suboptimal Mediterranean diet adherence, with particularly low intake of emblematic components such as fish, nuts, and olive oil—an important equity signal given that deprivation is itself associated with recurrent vascular risk [125].

Critically, the early post-event period appears to constitute a “teachable moment” in which structured nutritional input can measurably improve dietary trajectories: a monocentric before–after study of hospitalized ischemic stroke patients showed that implementing a systematic dietary intervention during admission (versus discretionary counseling) produced significantly greater improvements in global dietary scores at 6 months, including higher fruit/vegetable and unsaturated fat scores, thereby demonstrating that adherence-related behaviors can be shifted by embedding dietetics into acute stroke unit workflows—although effects on recurrent stroke and cardiovascular events were not assessed and remain a priority for future trials [126].

Randomized evidence in recent AIS survivors further supports feasibility and short-term adherence gains. In the ADD-SPISE Phase 2 randomized trial (2018–2022), 200 patients within one month of ischemic stroke were assigned to an avocado-adapted Mediterranean diet or a low-fat diet for 90 days, with repeated dietitian contacts and adherence monitoring (including the 14-item Mediterranean Diet Adherence Screener, MEDAS). The Mediterranean arm achieved significant improvements in adherence (including an approximately four-point increase in MEDAS), and the intervention was safe and acceptable; however, LDL-cholesterol at 90 days did not differ significantly between groups, plausibly reflecting high background use of high-intensity statins and improvements in both dietary arms (i.e., a “floor effect” on LDL lowering) [127].

This pattern is clinically instructive for secondary prevention: the principal value of post-stroke Mediterranean diet adherence may lie less in incremental LDL reduction beyond contemporary lipid-lowering pharmacotherapy, and more in its multi-domain impact on cardiometabolic and vascular phenotypes that are incompletely addressed by single-agent medications (e.g., blood pressure regulation, weight/waist control, glycemic stability, inflammatory milieu, and overall diet quality). Consistent with this broader framing, a systematic review of experimental studies in post-stroke adults identified only six eligible intervention trials up to August 2021 but concluded that Mediterranean diet interventions appear beneficial for systolic/diastolic blood pressure, LDL cholesterol, body mass index, and waist circumference—intermediate endpoints with established relevance to recurrent cerebrovascular risk—while underscoring substantial heterogeneity in intervention definitions and the need for more robust, stroke-specific randomized trials with hard outcomes [110].

Indeed, high-level syntheses caution against overstatement: in a Lancet Neurology appraisal, the authors emphasized that, although Mediterranean-style diets are consistently associated with lower first-stroke risk, the relationship between diet quality and recurrent stroke is less certain and reliable randomized evidence demonstrating recurrence reduction remains limited, reflecting both methodological challenges and historical underinvestment in dietary trials in secondary stroke prevention [128].

Against this evidentiary backdrop, an implementation-oriented interpretation is warranted: adherence to the Mediterranean diet after AIS is strongly justified on pathophysiological and risk-factor grounds and is guideline-endorsed; however, achieving sustained adherence requires interventions that explicitly accommodate stroke sequelae and the social context. Promising strategies include systematic inpatient dietetic consultation (to capitalize on early motivation), repeated reinforcement post-discharge (telephone/video follow-up, caregiver inclusion), and co-designed programs tailored to disability profiles and access barriers—approaches exemplified by structured, stakeholder-informed Mediterranean diet interventions developed specifically for stroke survivors and delivered via telehealth to improve accessibility and individualization [129].

## 7. Conclusions

Overall, the evidence presented in this review support the fact that the Mediterranean diet is much more than a cultural tradition—it is a clinically proven lifeline for cerebrovascular health. By targeting and reducing the main modifiable risk factors for ischemic stroke—such as high blood pressure, abnormal cholesterol, insulin resistance, systemic inflammation, endothelial dysfunction, and thrombosis—the diet offers a broad preventive benefit unmatched by any single drug therapy.

Furthermore, the advantages of following this dietary approach extend beyond lowering the risk of cardiovascular and cerebrovascular events; they also influence prognosis and post-stroke recovery. This represents a substantial shift in how chronic cerebrovascular disease is managed, highlighting not only the importance of antiplatelet drug prophylaxis but also the need for ongoing attention to vascular health, including dietary strategies that can reduce residual disability.

Randomized trials such as PREDIMED and its extensions show clear reductions in stroke incidence, while mechanistic and biomarker studies clarify the molecular pathways—from increased nitric oxide bioavailability to suppression of the NLRP3 inflammasome—by which these results are achieved (Figure 4). Research further demonstrates that culturally adapted menus, validated adherence tools, and digital coaching platforms can translate this evidence into sustainable, real-world practice. Together, these findings establish the Mediterranean diet as a foundation for both primary and secondary prevention of cerebrovascular disease. Future research should focus on refining precision-nutrition strategies by integrating genomics, metabolomics, and microbiome profiles to personalize dietary recommendations. There are several pilot studies and secondary analyses of the PREDIMED trial (including PREDIMED-Plus) that have examined metabolomics and microbiome signatures in relation to cardiovascular outcomes. Although large-scale prospective integration is rare, preliminary metabolomic profiles associated with adherence to the Mediterranean diet and stroke risk have been published. This is a concrete step towards “precision nutrition”, although there is still a long way to go in this regard. At the same time, public health policies should prioritize accessibility and affordability, ensuring that the benefits of this dietary pattern are accessible to all socioeconomic groups. Adopting the Mediterranean diet thus provides a practical, evidence-based approach to reducing the global burden of cerebrovascular disease and promoting healthy vascular aging. But, at the end of it all, can genetic polymorphisms predict dietary response in stroke patients? We still need many answers regarding the interaction between micronutrients and host factors such as single gene polymorphisms in determining their bioavailability and thus in promoting post-stroke neurological recovery. Scientific research in this area is ongoing and fervent, to obtain the answers that we still do not have today and that we consider essential in order to make informed recommendations on the most suitable diet for each patient’s characteristics.

## 8. Limitations

As this paper is a narrative review, it has the limitation of not having considered other datasets in addition to the PREDIMED trial cohort, such as the MESA or EPIC cohorts; the reason for this exclusion is that the considerations made, particularly regarding cerebrovascular risk and recovery after the acute phase of ischemic stroke, would introduce too much heterogeneity in people of different countries: as authors of this manuscript, we are aware that the effects of the MedDiet may vary in non-Mediterranean populations (e.g., due to different basic dietary habits or genetic backgrounds).

## Figures and Tables

**Table 1:** Summary of the relationship between the Mediterranean diet and ischemic stroke in terms of event risk and prognostic trajectory. (EVOO: extra-virgin olive oil; CRP: C-reactive protein; IL-6: Interleukin-6; BBB: blood–brain barrier; ↑: increase; ↓: decrease.

Med Diet and Ischemic Stroke	Key Findings	Protective Mechanisms
primary prevention	PREDIMED (2013, n = 7447): −30% cardiovascular events; −46% risk of stroke with MedDiet + EVOO	↓ inflammation (CRP, IL-6), ↓ platelet aggregation, ↑ endothelial function
high adhesion vs. low adhesion	Meta-analysis [121], n = various, >10,000: −17–22% risk of stroke	Improved lipid profile, ↓ blood pressure, ↑ insulin sensitivity
post-stroke recovery	[122] Better functional outcome (modified Rankin Score, Barthel Index) with MedDiet	Neuroprotective nutrients (omega-3, polyphenols) → ↓ neuroinflammation, ↑ neurogenesis
PREDIMED-Plus analysis	PREDIMED-Plus (2019, n = 6874): Longer telomeres, less inflammation, better body composition	Effects on cellular aging, microbiota, metabolism
preclinical studies	Rats with MedDiet (preclinical, n = various, 2015): −30% infarct volume, improved motor recovery	BBB protection, ↓ oxidative stress, ↓ pro-inflammatory cytokines

## Data Availability

Data sharing is not applicable to this article as no new data were created or analyzed in this study, as this is a narrative literature review.
